# Beloved Physicians

**Published:** 1881-06

**Authors:** W. R. Monroe

**Affiliations:** Baltimore


ARTICLE III.

### BELOVED PHYSICIANS.

BY W. R. MONROE, M. D., BALTIMORE.

> " The power of words and soothing sounds, appease
> The raging pain, and lessen the disease."

Human sympathy is more valuable in some cases than medicines. A knowledge of the healing art, or of the science of medicine is not all that is requisite to constitute a successful practitioner. Those tender feelings that spring from the heart, which constitute gentleness and goodness are not the result of intellectual culture alone, but rather of heart culture, and are supernatural. They spring from a higher source than human. Hence, an unrighteous, hard-hearted physician, or a skeptic in the Christian religion, lacks that love and sympathy at the bedside of his patient, which so essentially contributes to his comfort, and may hasten his recovery. The Great Physician has unerringly declared that "a good man out of the good treasure of his heart bringeth forth that which is good; and an evil man out of the evil treasure of his heart bringeth forth that which is evil." How, then, can a depraved and unregenerate hearted practitioner administer that godly consolation so often needed in the sick room?

> " Shame on those hearts of stone, that cannot melt
> In soft adoption of another's sorrow."

History confirms the idea of the sacred functions of the medical practitioner. The writings of Moses constitute a precious monument for the history of medicine; for they embrace hygienic rules of the highest sagacity, which, for their ability (apart from the religious ceremonies) would suit more modern times. Moses always gave his instructions concerning leprosy and other infirmities to the priests only; from which it may be safely inferred the Levites joined the practice of medicine to their sacerdotal functions. For many centuries they maintained this double relationship to society.

Josephus, the great historian, says: "God gave to Solomon a perfect knowledge of all the productions of nature, so that he compounded remedies, extremely useful, some of which had even the virtue to cast out devils."

M. Bouillaud, insists on the necessity of joining moral qualities to knowledge in the practice and teaching of medicine. He clearly demonstrates, that in default of morality, the most beneficent act is only an instrument of deception, and a dangerous weapon, in unsafe hands. He defines the true physician to be an honest man, instructed in the art of healing. *Vir probus medendi peritus.*

It is said that Hippocrates gained universal approbation, as much by his virtues as his genius. These virtues shine with great eclat in the clinical observations he has transmitted to us. He labored not for a reputation, but with the sole desire to be useful to his fellows in enlightening them on the best means of preserving health and in curing diseases.

Guy de Chauliac, the most famous physician and surgeon in Christendom, about the middle of the fourteenth century, in tracing the character of a surgeon, he recommends that he be learned, expert, ingenious; that he be bold when he is sure, and timid when in doubt; that he avoid bad cures and bad practices; be gracious to the sick; generous to his companions; wise in predictions; chaste, sober, pitiful, and merciful; not covetous, nor an extortionate of money, but receive a moderate fee, according to his labor, the abilities of his patient, the character of the issue or event, and his own dignity." M. Malgaigne, exclaims: "Never, since Hippocrates, has medicine heard a language stamped with such nobility and so few words."

Of Luke, "the beloved physician," but little is said. He is spoken of but three times in the Bible, yet he is the acknowledged author, by the universal church, of two of the most valuable books in the New Testament. How little we know of Luke as a man! Not a speech of his is extant! but deeds of silent men are often wiser than words from others. He was the companion of St. Paul. Luke lived, and listened, and wrote, while Paul appears to have done the talking.

A late writer has said that "a physician's career is remarkable for two things: for the unusual chance it furnishes of being useful, and for the large amount of unrequited, and even unappreciated, labor it demands. Brave and good doctors well deserve the epithet of "beloved." They are among the most devoted, the most patient, and the most generous people in the world. How cheerfully and promptly they give their nights and their days to those who call them, whether they have any rights or not! No newspapers praise them; the community has no words of admiration or cheer for half they do; they are underpaid, and often not paid at all. Yet they go on their quiet way. God never forgets them. The celebrated Boerhaave says: "My poor are my best patients; God pays for them." Some physicians pray with their patients, some actually sing gentle hymns with them at their bedside; some read to them or converse about the things of the Kingdom; some bring a hundred messages to the pastors concerning needs. Oh! scores of invalid people thank Heaven daily for their "beloved physicians."

The biographer of the late Dr. William Hauser, of Georgia, once a professor in the Oglethorpe Medical College at Savannah, and at that time an associate of the senior editor of this journal in the same institution, says of him: "When in active medical practice he invariably made it a rule to pray with his patients before he prescribed for them. No matter how sick the patient was, he would always kneel by the bedside and ask God for his blessing on the sick one before administering a dose of medicine."

"Speak gently! 'tis a little thing
Dropped in the heart's deep well;
The good, the joy that it may bring,
Eternity shall tell."

